# Diseases of the Throat, Nose and Ear

**Published:** 1913-12

**Authors:** 


**Diseases of the Throat, Nose and Ear.** By W. G. Porter, M.B., B.Sc. Pp. xii, 275. Bristol: John Wright & Sons Ltd. 1912. Price 7s. 6d. net.

In the preparation of such a work for the student and general practitioner the author is always faced by the great difficulty of deciding what should be omitted and what retained. On this occasion the effort has been eminently successful. Although only a small book of some 270 pages, the practitioner will find that none of the essentials in diagnosis and treatment, which lie within his sphere, have been omitted. The facts are clearly stated, and while the newest work is introduced, it is always easily understood. The more important lesions are represented in some excellent coloured plates prepared for this work. The pre-eminent importance of indicating the earliest danger signals in such serious conditions as malignant disease of the larynx and the intracranial complications of middle-ear disease is recognised and fulfilled.

Both author and publishers are to be congratulated on the production of a work which should supply a decided want.

